# Effects of therapeutic plasma exchange on the endothelial glycocalyx in septic shock

**DOI:** 10.1186/s40635-021-00417-4

**Published:** 2021-11-24

**Authors:** Klaus Stahl, Uta Carola Hillebrand, Yulia Kiyan, Benjamin Seeliger, Julius J. Schmidt, Heiko Schenk, Thorben Pape, Bernhard M. W. Schmidt, Tobias Welte, Marius M. Hoeper, Agnes Sauer, Malgorzata Wygrecka, Christian Bode, Heiner Wedemeyer, Hermann Haller, Sascha David

**Affiliations:** 1 Department of Gastroenterology, Hepatology and Endocrinology, Hannover Medical School, Carl-Neuberg-Str.1, 30625 Hannover, Germany; 2 Department of Nephrology and Hypertension, Hannover Medical School, Hannover, Germany; 3 Department of Respiratory Medicine and German Centre of Lung Research (DZL), Hannover Medical School, Hannover, Germany; 4 Department of Biochemistry, University Medicine Giessen, Giessen, Germany; 5 Department of Anaesthesiology and Intensive Care Medicine, University Hospital Bonn, Bonn, Germany; 6 Institute for Intensive Care Medicine, University Hospital Zurich, Zurich, Switzerland

**Keywords:** Extracorporeal treatment, DAMP, Plasmapheresis, Heparan sulfate, Heparanase

## Abstract

**Background:**

Disruption of the endothelial glycocalyx (eGC) is observed in septic patients and its injury is associated with multiple-organ failure and inferior outcomes. Besides this biomarker function, increased blood concentrations of shedded eGC constituents might play a mechanistic role in septic organ failure. We hypothesized that therapeutic plasma exchange (TPE) using fresh frozen plasma might influence eGC-related pathology by removing injurious mediators of eGC breakdown while at the time replacing eGC protective factors.

**Methods:**

We enrolled 20 norepinephrine-dependent (NE > 0.4 μg/kg/min) patients with early septic shock (onset < 12 h). Sublingual assessment of the eGC via sublingual sidestream darkfield (SDF) imaging was performed. Plasma eGC degradation products, such as heparan sulfate (HS) and the eGC-regulating enzymes, heparanase (Hpa)-1 and Hpa-2, were obtained before and after TPE. A 3D microfluidic flow assay was performed to examine the effect of TPE on eGC ex vivo. Results were compared to healthy controls.

**Results:**

SDF demonstrated a decrease in eGC thickness in septic patients compared to healthy individuals (*p* = 0.001). Circulating HS levels were increased more than sixfold compared to controls and decreased significantly following TPE [controls: 16.9 (8–18.6) vs. septic patients before TPE: 105.8 (30.8–143.4) μg/ml, *p* < 0.001; vs. after TPE: 70.7 (36.9–109.5) μg/ml, *p* < 0.001]. The Hpa-2 /Hpa-1 ratio was reduced in septic patients before TPE but normalized after TPE [controls: 13.6 (6.2–21.2) vs. septic patients at inclusion: 2.9 (2.1–5.7), *p* = 0.001; vs. septic patients after TPE: 13.2 (11.2–31.8), *p* < 0.001]. Ex vivo stimulation of endothelial cells with serum from a septic patient induced eGC damage that could be attenuated with serum from the same patient following TPE.

**Conclusions:**

Septic shock results in profound degradation of the eGC and an acquired deficiency of the protective regulator Hpa-2. TPE removed potentially injurious eGC degradation products and partially attenuated Hpa-2 deficiency.

*Trial registration* clinicaltrials.gov NCT04231994, retrospectively registered 18 January 2020

**Supplementary Information:**

The online version contains supplementary material available at 10.1186/s40635-021-00417-4.

## Take-home message

Septic shock results in profound degradation of the endothelial glycocalyx (eGC) and an acquired deficiency of the protective regulator Heparanase-2 (Hpa-2). Therapeutic plasma exchange removed injurious eGC degradation products and partially attenuated Hpa-2 deficiency.

## Background

Sepsis is a clinical syndrome characterized by life-threatening organ dysfunction caused by a dysregulated host response to infection. Septic shock features hypotension refractory to volume resuscitation and serum lactate elevation [1] commonly ending in death [2]. It has been recognized that global endothelial dysfunction, in particular breakdown of the vascular barrier, represents a cornerstone in the development of multi-organ failure in sepsis [3]. The endothelial glycocalyx (eGC), a gel-like structure of glycosaminoglycans and proteoglycans covering the entire luminal surface of the endothelium, contributes to the maintenance of vascular hemostasis including tone and permeability as well as inflammation and coagulation [4]. Disruption of this highly dynamic structure is observed early in sepsis, and the resultant eGC injury is strongly associated with later multi-organ failure and inferior outcomes [5–7]. Heparanase-1 (Hpa-1) is the enzyme primarily responsible for the injurious degradation of the major eGC component, heparan sulfate (HS), and is upregulated in sepsis [8]. By contrast, Heparanase-2 (Hpa-2), a protein that has been described as a protective antagonist of Hpa-1 [9, 10], is suppressed in murine sepsis models [11], suggesting imbalance of eGC-regulating proteins in sepsis. Furthermore, increased levels of shedded HS degradation products into the circulation are not only biomarkers of glycocalyx injury, but also can act as disease mediators, namely the so-called *Damage associated molecular patterns* (DAMPs), directly contributing to sepsis-associated organ failures such as cardiomyopathy [12] and encephalopathy [13]. Therapeutic efforts against sepsis morbidity and mortality are limited to anti-infectious measures (including antimicrobials and surgical or interventional focus sanitation) and organ support [14]. So far, no specific intervention targeting endothelial dysfunction, including eGC degradation, exists to the present time. Therapeutic plasma exchange (TPE) has been recently investigated as a potential adjunctive treatment strategy in early and severe septic shock [15]. The observed positive effects in rapid hemodynamic stabilization [15] could be related to removal of deleterious components or replacement of protective plasma proteins consumed by the disease process [16, 17].

Here, we hypothesize that TPE against plasma from healthy donors might (1) remove products of eGC shedding that serve as injurious DAMPs (e.g., HS) and (2) simultaneously compensate deficiency of protective Hpa-2 to attenuate imbalance of eGC regulation enzymes. In this study, we therefore investigated the effect of a single TPE against fresh frozen plasma (FFP) on key constituents of the eGC as well its regulating enzymes in the blood of patients with early and severe septic shock. Additionally, quantitative sublingual sidestream darkfield (SDF) imaging in vivo as well as microvascular perfusion studies in an ex vivo endothelial microfluidic chip model, were undertaken to test our hypothesis.

## Methods

### Study population

**Fig. 1:**
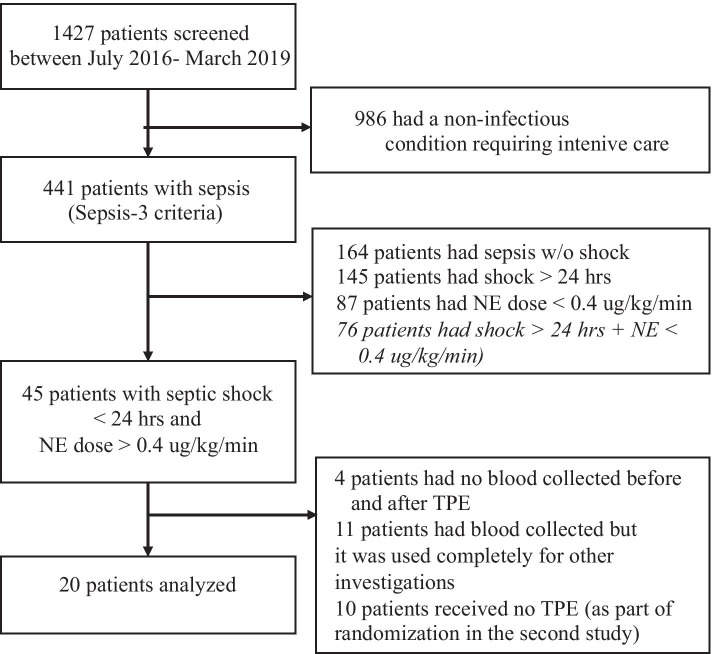
Flowchart of study participants. NE means norepinephrine and TPE therapeutic plasma exchange

This study was a post hoc analysis from a subset of patients included in both a single-center non-randomized study [15] and a recently concluded randomized study (NCT04231994, accepted for publication, unpublished). Data and bio-samples were acquired from patients receiving TPE directly before and after the TPE procedure and results were further compared to healthy individuals used as controls. In total, we screened 1.427 patients submitted to our 14-bed medical ICU from July 2016 to March 2019 for the presence of sepsis using the SEPSIS-3 definition [1]. Of the 45 patients included in both studies, we finally analyzed a subgroup of 20 patients, all of whom received additional TPE treatment and provided sufficient blood sample volumes for all further analysis steps (Fig. 1). All patients were treated according to the 2016 Surviving Sepsis Campaign (SSC) guidelines. Patients were included based on: (i) septic shock with need for vasopressors < 24 h prior to entry, and (ii) profound systemic hypotension requiring norepinephrine (NE) doses of > 0.4 µg/kg/min despite adequate intravenous fluid resuscitation (≥ 30 ml/kg bodyweight crystalloids). As exclusion criteria, we defined unavailability of TPE within first 6 h after study inclusion, pregnancy or breast feeding, age < 18 years, end-stage chronic disease, and presence of a directive to withhold life-sustaining treatment. The ethical committee of Hannover Medical School approved both study protocols (EK 2786-2015 and EK 8852_MPG_23b_2020) and written informed consent was obtained from participants or authorized representatives. The study was performed in accordance with the ethical standards laid down in the 1964 Declaration of Helsinki and its later amendments. Demographic and clinical data were obtained immediately at study inclusion before TPE. For comparison, following informed consent, 20 healthy controls without pre-existing medical conditions were included into the study.

### TPE

Vascular access was established by venous insertion of an 11-French two-lumen hemodialysis catheter. TPE was performed in a singular treatment session against a fixed dose of 12 units of FFP. All patients received single shot triple anti-allergic prophylaxis with 100 mg decortin, 2 mg clemastine (H1 inhibitor) and 50 mg ranitidine (H2 inhibitor), given intravenously directly before start of the TPE treatment. Anticoagulation during TPE was achieved by regional citrate infusion. 11/20 (55%) of patients received additional prophylactic anticoagulation by continuous low dose (median (IQR) 400 (400–800) IU/h) heparin. In patients with acute kidney injury (AKI), hemodialysis was interrupted for the duration of TPE. Blood samples were drawn before and after TPE. NE dose was titrated every 10–15 min to maintain a mean arterial pressure (MAP) above 65 mmHg.

### Data and sample collection

Patient plasma and serum blood samples were acquired before and after TPE and therefore time elapsed between both blood draws was short (3 ± 1.5 h). For comparison, we used plasma and serum from healthy human donors without pre-existing serious medical conditions after written informed consent. Plasma samples were collected in non-heparinized tubes. No personal or identifying information were collected from study participants. All samples were stored at – 80 °C until use. All personal patient data were anonymized before further analysis. Data were collected using electronic medical records including the patient data monitoring system (PDMS) m.life (Version 10.5.0.71, medisite GmbH, Berlin, Germany). Sequential Organ failure Assessment (SOFA) scores were calculated according to the description by Vincent et al. [18].

### Assessment of the eGC thickness in vivo

The eGC thickness was assessed non-invasively using a SDF camera (CapiScope HVCS, KK Technology, Honiton, UK) coupled with the GlycoCheck™ software. The software automatically detects microvessels with diameters between 5 and 25 µm and records eGC thickness, determined by analysis of the dynamic lateral movement of red blood cells into the permeable part of the eGC layer expressed as the *Perfused boundary region* (PBR) (in µm). All measurements were performed at the time of blood sampling before initiation of TPE by observers experienced in the method as described before [6]. Three consecutive measurements were taken and then averaged.

### Enzyme-linked immunosorbent assays

ELISAs specific for Heparanase-1 (amsbio, #EA1340Hu) and Heparanase-2 (amsbio, #E5389Hu) were performed in plasma samples. All samples were measured using a Tecan absorbance microplate reader (Sunrise, #F039300) at 450 nm. All measurements were performed in duplicates. Hyaluronan concentration was measured using the Hyaluronan DuoSet ELISA according to manufacturer’s instruction (R&D Systems, Wiesbaden, Germany).

### Glycosaminoglycan isolation and purification

The plasma samples were diluted 1:1 in 0.1 M K_2_HPO_4_ (pH 8.0) and then subjected to proteolysis with 100 µg/ml proteinase K (Sigma-Aldrich, Darmstadt, Germany) at 56 °C for 24 h. Afterwards, the samples were heated at 90 °C for 10 min and filtrated on Ultrafree-MC filters (0.22 µm membrane) at 12,000*g* for 10 min. Filtrates were recovered, mixed with equal amounts of 1.5% sodium nitrate and 33% of acetic acid, and incubated at room temperature with gentle shaking for 1 h. The reaction was stopped by the addition of 12.5% ammonium sulfate (half of a total sample volume). Subsequently, the samples were transferred to the tubes containing saline and Triton X-100 (final concentration 1%), vigorously shacked at room temperature for 30 min and then cooled down to 4 °C using ice bath. Finally, the samples were supplemented with trichloroacetic acid to the final concentration of 10% at incubated at 4 °C for additional 10 min, followed by centrifugation at 12.000*g* for 10 min and washing with chloroform. The aqua phase was dialyzed (3500 MWCO) against buffer containing 40 mM ammonium acetate and 3 mM CaCl_2_ (pH 6.8) for 16 h. The glycosaminoglycans were collected and either directly subjected to digestion or lyophilized.

### Enzymatic digestion of glycosaminoglycans and liquid chromatography–mass spectrometry (LC–MS) analysis

The glycosaminoglycans were digested with 0.1 U/ml chondroitin lyase ABC (Sigma-Aldrich, Darmstadt, Germany) in 40 mM ammonium acetate and 3 mM CaCl_2_ (pH 6.8) at 37 °C overnight. The digestion products were recovered by centrifugal filtration (3500 MWCO) and freeze-dried for LC–MS analysis. Alternatively, glycosaminoglycans were treated with 1 U/ml of heparinase enzymes (Iduron, ALderley Edge, United Kingdom) at 30 °C for 10 h or with 60 U/ml hyaluronidase (Sigma-Aldrich) 37 °C for 6 h. Derivatization of unsaturated disaccharides with 2-aminoacridone (AMAC) and LC–MS analysis were performed as previously described [19].

### Microfluidic experiments

Microfluidic chips were fabricated of polydimethylsiloxane (PDMS) (Sylgard 184, Dow Corning) by replication of the polymeric master as described by our group previously [11]. Naive human microvascular endothelial cell (HMEC-1) line (ATCC) was incubated in MCDB131 medium under flow conditions (38.75 dyn/cm^2^) for 5 days before the experiments. Then, cells were treated with medium supplemented with 10% serum from one representative patient (collected before and directly after TPE and taken directly to the perfusion system) or control serum (pooled from four healthy controls) for 6 h under flow conditions. The cells were fixed with 4% PFA under flow. Then, chips were disconnected from the flow and cells were permeabilized with 0.1% Triton X-100/PBS and stained using anti-heparan sulfate antibody (Amsbio, 10E4260917, dilution: 1:100) and as a secondary antibody for confocal microscopy anti-mouse-alexa 633 (ThermoFisher Science, A21056, dilution: 1:500). Confocal microscopy was then performed without flow using Leica TCS-SP2 AOBS confocal microscope (Leica Microsystems) at the Core Facility for Laser Microscopy at Hannover Medical School. All the images were taken with oil-immersed ×63 objective, NA 1.4. Series of z-scans were processed and quantified using ImageJ software. 3D reconstruction of the eGC structure was performed using Leica LasX software.

### Statistical analysis

Data were presented as median with interquartile range (IQR). Two-tailed *p* values of less than 0.05 were considered to indicate statistical significance. Paired *t*-test or Wilcoxon test (as appropriate) was utilized in order to compare longitudinal values before (pre) and after (post) TPE. Unpaired *t*-test and Mann–Whitney test (for not normal distributed variables) were employed to compare unpaired values. We used GraphPad Prism 7 (Graph Pad, La Jolla, CA, USA) and SPSS Statistics Version 25 (SPSS Inc., Chicago, IL, USA) for data analysis and graph generation.

## Results

### Cohort characterization

Demographic and clinical details are summarized in Table 1. Eighty percent of the patients were men, and the median (IQR) age was 53 (34–59) years. Lungs and the abdomen were the most common sites of infection. A causative pathogen was identified in 65% of the cases. Gram negative and positive pathogens were most commonly identified and in 10% more than one pathogen was detected. All patients were treated with a combination of broad-spectrum antibiotics. Median (IQR) SOFA score was 18 (15–19). All patients were in severe shock indicated by high NE doses and lactate concentrations at study inclusion. Patients displayed signs of severe hyper-inflammation as indicated by high levels of C-reactive protein (CRP) and procalcitonin (PCT). Ninety percent were mechanical ventilated and had an oxygenation index (PaO_2_/FiO_2_) of 130 (117–189). AKI with need for renal replacement therapy (RRT) was present in 65% of the patients at time of inclusion.

**Table 1:** Demographic and clinical characteristics at baseline

Category	Median (IQR)/*n* (%)
Age—year	53 (34–59)
Sex—no (%)	
Male	16 (80)
Female	4 (20)
BMI—kg/m^2^	27.8 (21.9–32.6)
Sepsis onset—no (%)	
Ambulatory	14 (70)
Hospital acquired	6 (30)
Side of infection—no (%)	
Lung	14 (70)
Abdomen	3 (15)
Urogenital	1 (5)
Soft tissue	1 (5)
More than one	1 (5)
Identified pathogen—no (%)	
Gram+	3 (15)
Gram−	6 (30)
Fungi	1 (5)
Viral	1 (5)
More than one	2 (10)
Non-identified	7 (35)
SOFA	18 (15–19)
Norepinephrine dose (μg/kg/min)	0.734 (0.564–1.206)
CRP (mg/l)	267 (151–325)
PCT (μg/l)	24 (9–99)
Lactate (mmol/l)	6.6 (2.6–11.3)
Mechanical ventilation—no (%)	18 (90)
Oxygenation index (PaO_2_/FiO_2_)	130 (117–189)
Renal replacement therapy—no (%)	13 (65)

Given are demographic and clinical characteristics at the time of study inclusion before therapeutic plasma exchange (TPE) treatment. Values are presented as median (25% to 75% interquartile range) or if categorical as numbers and percentages. Demographic characteristics for the control patients were: median (IQR) age 50 (28–63) years and 15/20 males for laboratory investigation controls; 39 (36–44) years and 5/10 males for SDF measurement controls. All individuals of the control cohorts had no relevant medical preconditions

*BMI* body mass index, *SOFA* Sequential Organ Failure Assessment, *CRP* C-reactive protein, *PCT* procalcitonin

### Assessment of endothelial glycocalyx thickness in vivo

**Fig. 2:**
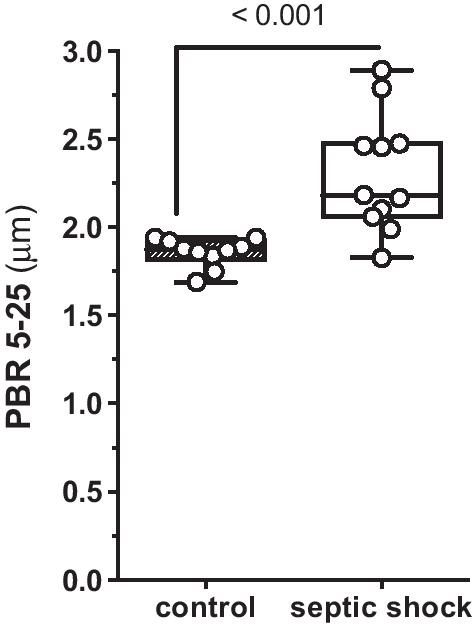
Injury to the endothelial glycocalyx (eGC) in vivo. Sublingual dark field imaging in patients allows quantification of the eGC thickness as indicated by the perfused boundary region (PBR). Box and whisker plots showing results for healthy controls (*n* = 10) as well as patients with septic shock before commencement of therapeutic plasma exchange (TPE) (*n* = 11)

We quantified the size of the individual patients’ eGC in the sublingual microvasculature using an indirect surrogate termed PBR. The larger the PBR (i.e., the diameter where the blood cells move in a micro-vessel), the smaller the thickness of the eGC. We found (Fig. 2) a decrease in the thickness of the eGC in septic patients compared to healthy individuals indicated by increased PBRs [median (IQR) PBR for controls: 1.88 (1.82–1.93) μm vs. septic patients at inclusion: 2.18 (2.06–2.48) μm, *p* = 0.001]. In a representative video recording, almost complete stasis of microvascular blood flow can be seen at study inclusion, which was improved in a repetitive recording directly following TPE treatment (Additional file 1: Video S1 and Additional file 2: Video S2).

### Effect of TPE on products of endothelial glycocalyx shedding

**Fig. 3:**
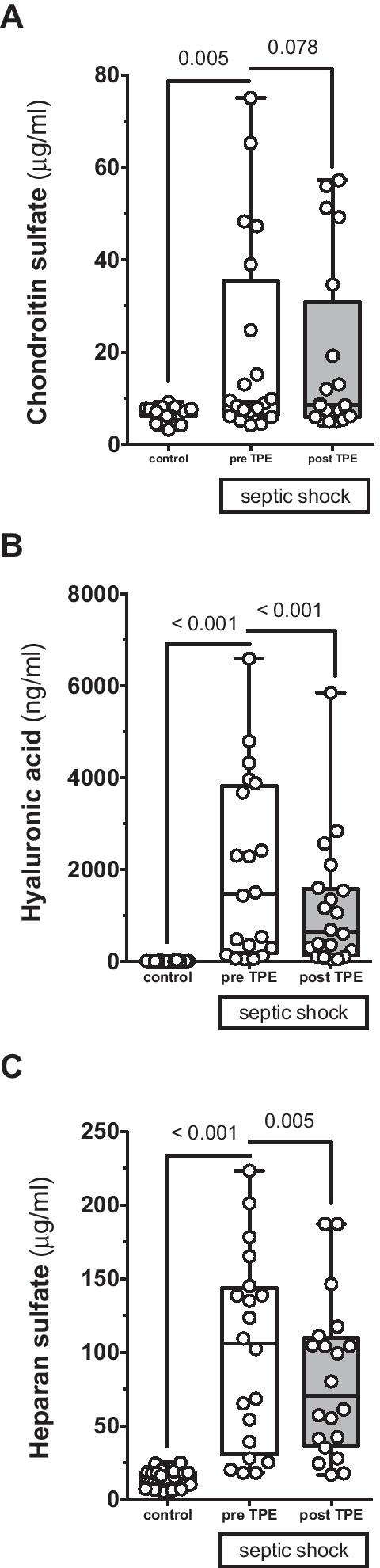
Effect of therapeutic plasma exchange (TPE) on glycocalyx shedding products. Box and whisker plots showing blood concentrations of hyaluronic acid (HA) (**A**), chondroitin sulfate (CS) (**B**) and heparan sulfate (HS) (**C**) for healthy controls (*n* = 20) as well as patients with septic shock before (pre) (*n* = 20) and directly after (post) (*n* = 20) TPE

Three key constituents of the eGC were investigated in the patients’ blood employing mass spectroscopy analysis (Fig. 3). Chondroitin sulfate (CS) concentrations (Fig. 3A) were increased at study inclusion compared to healthy controls but were not significantly reduced following TPE (controls: 7.1 (6.3–7.7) μg/ml vs. septic patients at inclusion: 9.3 (6.5–35.5) μg/ml, *p* = 0.005; vs. septic patients after TPE: 8.5 (6–30.8) μg/ml, *p* = 0.078). HA (Fig. 3B) was increased in septic individuals and reduced by more than half following TPE (controls: 10.1 (5.8–17.4) ng/ml vs. septic patients at inclusion: 1476 (188.3–3834) ng/ml, *p* < 0.001; vs. septic patients after TPE: 652.9 (146.1–1592) ng/ml, *p* < 0.001). Comparable, HS (Fig. 3C) concentrations were increased more than six times compared to controls and were decreased by about a third following a single TPE treatment (controls: 16.9 (8–18.6) μg/ml vs. septic patients at inclusion: 105.8 (30.8–143.4) μg/ml, *p* < 0.001; vs. septic patients after TPE: 70.7 (36.9–109.5) μg/ml, *p* < 0.001).

### Effect of TPE on regulating enzymes of the endothelial glycocalyx

**Fig. 4:**
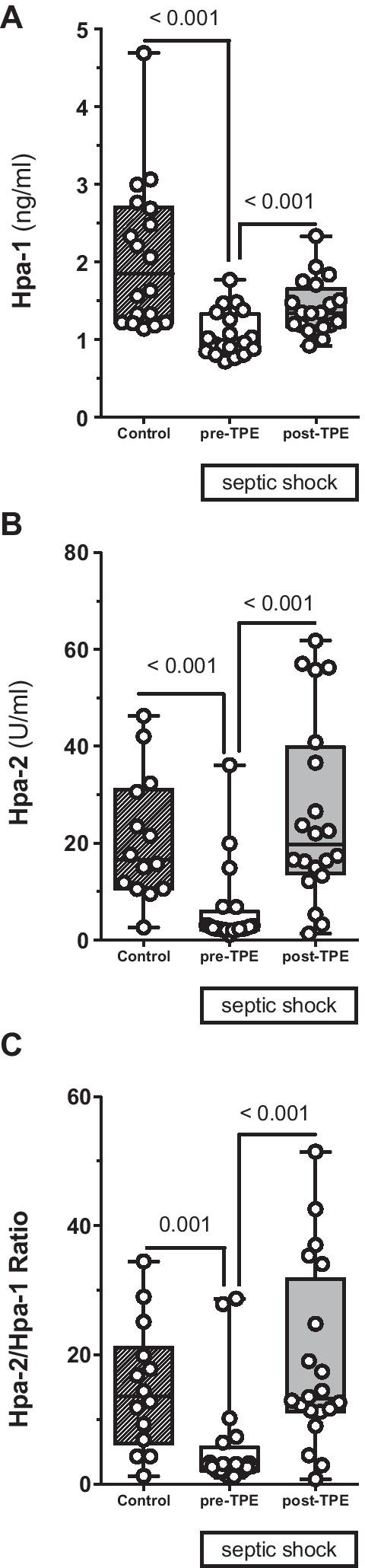
Effect of therapeutic plasma exchange (TPE) on key regulating enzymes of the endothelial glycocalyx (eGC). Box and whisker plots showing blood concentrations of circulating Heparanase-1 (Hpa-1) (**A**), Heparanase-2 (Hpa-2) (**B**) as well as the ratio of Hpa-2 to Hpa-1 (**C**) for healthy controls (*n* = 18) as well as patients with septic shock before (pre) (*n* = 20) and directly after (post) (*n* = 20) TPE

The key glycocalyx sheddase Hpa-1 (Fig. 4A) was decreased in septic shock patients compared to controls and increased following TPE [controls: 1.8 (1.2–2.7) ng/ml vs. septic patients at inclusion: 1 (0.9–1.3) ng/ml, *p* < 0.001; vs. septic patients after TPE: 1.3 (1.2–1.7) ng/ml, *p* < 0.001]. Even more noticeable, the Hpa-1 counterpart, Hpa-2 (Fig. 4B), was reduced by 85% in septic patients compared to non-septic individuals and was substituted by TPE treatment reaching normal blood concentrations following TPE [controls: 16.7 (10.6–31.1) U/ml vs. septic patients at inclusion: 2.5 (2.2–5.9) U/ml, *p* < 0.001; vs. septic patients after TPE: 19.7 (13.8–39.8) U/ml, *p* < 0.001]. Consequentially, the ratio of Hpa-2 to Hpa-1 (Fig. 4C) concentration was reduced in septic patients before TPE and normalized after TPE [controls: 13.6 (6.2–21.2) vs. septic patients at inclusion: 2.9 (2.1–5.7), *p* = 0.001; vs. septic patients after TPE: 13.2 (11.2–31.8), *p* < 0.001].

### In vitro proof-of-concept experiment using a microfluidic chip model

**Fig. 5:**
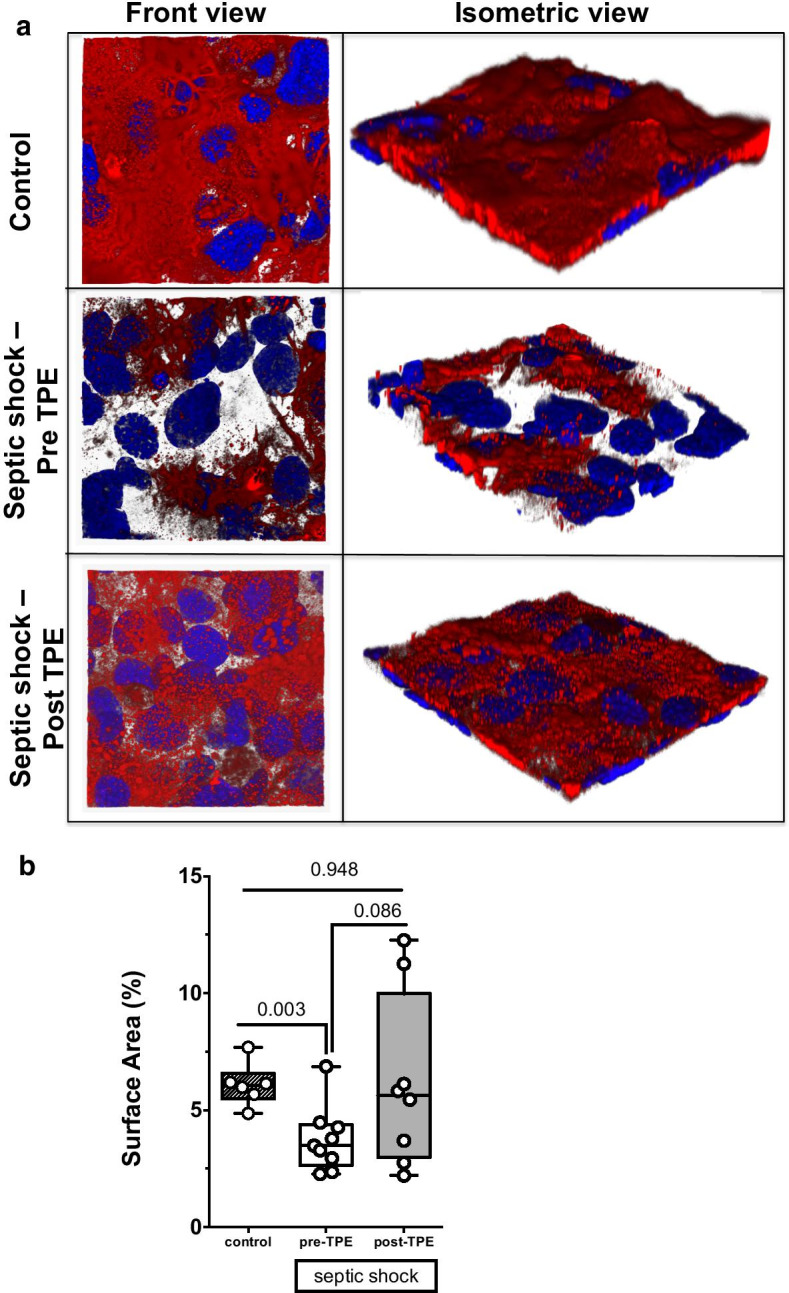
Ex vivo stimulation of 3D microvessels with septic serum before and after therapeutic plasma exchange (TPE). Exemplary 3D reconstruction of the heparan sulfate (HS) layer images of naive endothelial cells in a microfluidic chip (HS in red, DAPI nuclei staining in blue) after perfusion with serum and incubation overnight of a representative septic patient both before and after TPE compared to healthy control serum (pooled out of four control patients) (**A**) and quantification of the HS positive surface area as box and whisker plots (**B**)

To demonstrate that the degradation of the eGC induced by septic serum might be attenuated following TPE treatment, we used a microfluidic chamber with cultured endothelial cells under flow over 3 days that synthesize an intact and stable eGC under in vitro conditions. After stimulation with serum from a representative septic patient before and after TPE treatment or control serum, the eGC was visualized by confocal microscopy followed by computerized 3D reconstruction (Fig. 5A). The percentage of surface area covered with HS rich eGC is then quantified by analyzing the HS positive area. We found that stimulation with serum from a representative patient with septic shock before TPE was sufficient to severely damage the eGC indicated by a reduced HS positive area on the surface of endothelial cells (Fig. 5A). The HS positive area (Fig. 5B) was reduced by 37% compared to healthy controls (*p* = 0.003). Consistent with our hypothesis that TPE treatment replenishes the deficit in protective Hpa-2 in septic patients, we observed that loss of HS positive area on endothelial cells was attenuated following perfusion with serum collected from the same patient after receiving TPE (Fig. 5A). Quantitative analysis demonstrated a trend towards higher HS surface areas when stimulation with serum collected pre- (3.7 ± 1.4%) and after (6.2 ± 3.7%) TPE were compared (*p* = 0.086 for pre vs. post-TPE, *p* = 0.948 for post-TPE vs. control, Fig. 5B).

## Discussion

We investigated the effects of early TPE on the eGC in septic shock. Patients included in this investigation experienced a severe form of septic shock as indicated by high NE requirement, inflammatory markers and a high prevalence of multi-organ failure. We found that a single and early TPE had a profound effect on multiple different eGC-related parameters in these patients and report several relevant findings.

First, we could confirm a profound decrease in eGC thickness, indicating shedding of the eGC in septic patients compared to healthy controls by in vivo SDF measurement. PBR values in this cohort were in line with previous observations made in septic patients [20]. Of note, median PBR values were exclusively found in a range that was previously demonstrated to correlate with a poor survival in sepsis patients, namely a value above 1.97 μm [21].

Second, three key constituents of the eGC, notably CS, HA and HS, were increased in the circulation of septic shock patients and two of them (HA and HS) could be decreased by TPE. Among them, CS was only mildly increased, which is in line with previous observations [19, 22]. In contrast, both HA and HS were substantially elevated in patients with sepsis. Considering the severity of septic shock in this cohort, these results reproduce previous observations that have closely correlated both HA and HS concentration to the severity of multi-organ dysfunction and mortality [6]. HA and HS, beyond being biomarkers of disease severity, have further been suggested to possess direct pathophysiological significance. HA causes amplification of the pro-inflammatory response through Toll-like receptor-4 (TLR4) [23, 24]. HS aggravate systemic septic inflammatory reaction by increasing signaling through TLR4 [11, 25] and stabilization of interferon-gamma [26], potentially contributing to distant organ failure such as septic cardiomyopathy [12, 27]. Additionally, septic encephalopathy may be aggravated by direct binding of HS to protective neurotransmitters causing depletion of those within the central nervous system [13, 28]. Here, we could demonstrate that TPE might be an effective tool to rapidly eliminate circulating HA and HS in septic patients.

Third, TPE attenuated an acquired imbalance of essential eGC regulation enzymes. The process of eGC degradation—leading to subsequent appearance of toxic shedding products in the patients’ blood—is closely regulated by specific enzymes. We investigated whether or not TPE could also affect these underlying regulatory enzymes. As HS is the most abundant constituent of the eGC and its role as a toxic DAMP has been described most extensively, we chose to focus on HS. Hpa-1 is primarily responsible for degradation of HS chains, and its enzymatic activity was increased in septic pulmonary [5] and renal [7] failure. While some previous reports found the concentration of Hpa-1 increased [12] in the blood of septic patients, others observed reduced blood concentrations of Hpa-1 [29]. In our study, Hpa-1 concentrations were significantly reduced compared to healthy controls. Given the close binding of Hpa-1 within the HS rich layer eGC on inflammatory stimuli [30], one could speculate that a reduced Hpa-1 blood concentration might indicate an increased shift of Hpa-1 from the soluble compartment to the eGC layer leading to increased shedding of HS.

Previous observations found the protective antagonist, termed Hpa-2, to be depleted in septic mice blood and renal tissue [11]. Here, we confirm these pre-clinical findings for the first time in septic patients. An analogous role of acquired Hpa-2 deficiency in critical ill Covid-19 patients has been recently reported by our group [31]. In the present study, TPE did not only replenish deficient Hpa-2, but completely corrected the imbalanced ratio of protective Hpa-2 to injurious Hpa-1. We speculate that correcting the balance of regulating mediators of critical HS content might translate into improved eGC stability under septic conditions. This observation suggests that TPE might evolve its therapeutic potential in sepsis not only by removing injurious molecules but also by replacing consumed protective factors. A similar state-of-affairs has been demonstrated earlier for proteins implicated in anticoagulation [16, 17].

Finally, we applied a 3D ex vivo model using septic serum. This stimulus was sufficient to severely damage the eGC in a microfluidic chamber with cultured endothelial cells that synthesize an intact and stable eGC under in vitro conditions. Stimulation of this complex ex vivo cellular system with septic serum led to an acute degradation of HS. We suggest that this model represents an experimental correlate of the shedding exhibited by our patients. When we used serum from the same septic shock patients after TPE was performed, the eGC was protected in this in vitro model. These observations suggest that TPE might reduce eGC shedding by rebalancing the homeostasis between Hpa-1/Hpa-2.

Together, our data propose that TPE might indeed combine two important effects in one singular intervention—removing excessive injurious mediators (e.g., HS degradation products that serve as DAMPs) while at the same time replacing protective but consumed factors (e.g., Hpa-2). In line with these observations, we have elaborated on this two-edged concept [17] previously concerning factors of inflammation/anti-inflammation [15, 32], pro-/anticoagulation [16] as well as endothelial permeability/ant-permeability [15].

This study has several limitations, mainly its small sample size as well as the retrospective nature, and should therefore be assessed as primarily hypothesis generating. SDF measurement was only feasible in a subset of patients, mainly due to patient and caregiver specific limitations (e.g., prone positioning). This clearly might affect reliability of PBR measurement. The in vitro investigation demonstrated data from a singular exemplary patient since usage of fresh patient serum appears to be essential to yield eGC degradation following microfluidic experiments. In addition, the intervention was administered at a fixed dose, which precludes us from providing data on effects at different dosages or time frames. Given the post hoc nature of this analysis, longitudinal assessment of glycocalyx related parameters was not possible. For instance, PBR was not analyzed in the following days when de novo synthesis might have generated novel glycocalyx sugars. The restoration of a hydrodynamically relevant glycocalyx layer in vivo requires about 7 days [33]. It is therefore not to be expected that eGC thickness can improve within a short period of time. It has been demonstrated before, that certain heparan sulfate fragments induce septic encephalopathy in a length- and sulfation-dependent manner [13], however an additional in depth analysis of fragment length and sulfation pattern was not possible in this study due to shortage of probe volume. As no plasma effluent was collected, we cannot directly demonstrate active removal of eGC fragments by TPE. It may also be plausible that the shift towards “control” of eGC constituents and Hpa-1/Hpa-2 ratio is purely due to an equilibration of these levels within normal plasma (FFP levels of eGC constituents and Hpa-1/Hpa-2 levels) caused by TPE rather than a physiologic effect of TPE on Hpa-1/Hpa-2 ratios and a resultant reduction in shedding. As low dose heparin was administered in about half of the patients and heparin has been suggested to exert protective effects on the endothelial glycocalyx in pre-clinical investigations [5, 34], one could speculate on a confounding effect of heparin anticoagulation. However, HS concentrations were not different in patients with- and without heparin anticoagulation, neither before- or after TPE (data not shown). Furthermore, no data exist, investigating the effect of low dose heparin on the eGC in septic patients. All patients received single shot anti-allergic prophylaxis with 100 mg decortin given intravenously directly before start of the TPE treatment. Glucocorticoids have been used in the treatment of sepsis for more than four decades, mainly for its multiple anti-inflammatory and pleiotropic effects [35]. In fact, hydrocortisone has been associated in pre-clinical experiments with protection of the eGC, such as in ischemia reperfusion injury [36, 37] and following stimulation with TNF-α [38]. However, except a postulated protective effect of corticosteroids on the glomerular glycocalyx [39], no data exist on the impact of steroids on the eGC during sepsis and septic shock. On the other hand, adverse effects of corticosteroid use on glycocalyx structure and endothelial permeability have also been reported [40, 41]. Although our study cannot exclude an effect of pre TPE administered glucocorticoids on the eGC, this effect has yet to be better defined.

## Conclusions

In this prospective exploratory trial, severe septic shock was associated with increased eGC shedding and an acquired deficiency in protective Hpa-2. Early TPE treatment partially attenuated this imbalance by removing potentially injurious excessive shedding products while at the same time replacing the deficient eGC stabilizing enzyme Hpa-2. These data may not be generalizable to all patients with septic shock (i.e., with lower vasopressor doses) and not with less severe forms of sepsis. Whether or not an appropriately powered randomized controlled trial TPE might improve outcome in septic patients, remains to be determined.

## Supplementary Information

**Additional file 1: Video S1. **Representative video recordings of microvascular flow before and after Therapeutic Plasma Exchange (TPE). Sublingual dark field imaging video recordings visualizing microvascular flow conditions in a singular representative patient both before (Video S1) and directly after (Video S2) TPE treatment. The region of interest (marked with white bold arrows) demonstrates stasis of microvascular blood flow in a septic shock patient before TPE (Video S1), which is significantly improved in the same patient directly following TPE treatment (Video S2).

**Additional file 2: Video S2. **Representative video recordings of microvascular flow before and after Therapeutic Plasma Exchange (TPE). Sublingual dark field imaging video recordings visualizing microvascular flow conditions in a singular representative patient both before (Video S1) and directly after (Video S2) TPE treatment. The region of interest (marked with white bold arrows) demonstrates stasis of microvascular blood flow in a septic shock patient before TPE (Video S1), which is significantly improved in the same patient directly following TPE treatment (Video S2).

## Data Availability

The datasets used and analyzed are during the current study are available from the corresponding author on reasonable request.

## Abbreviations

AKI: Acute kidney injury
BMI: Body mass index
CRP: C-reactive protein
CS: Chondroitin sulfate
DAMPs: Damage associated molecular patterns
eGC: Endothelial glycocalyx
HS: Heparan sulfate
Hpa-1: Heparanase-1
Hpa-2: Heparanase-2
FFP: Fresh frozen plasma
HA: Hyaluronic acid
LC–MS: Liquid chromatography–mass spectrometry
MAP: Mean arterial pressure
NE: Norepinephrine
PBR: Perfused boundary region
PCT: Procalcitonin
RCT: Randomized controlled trial
RRT: Renal replacement therapy
SDF: Sublingual sidestream darkfield
SOFA: Sequential Organ Failure Assessment
TPE: Therapeutic plasma exchange
